# Supplementary material for: Village-level surveillance of neonatal disease with integrated real-time dashboards and quality-control in Uganda
Source: medRxiv. 2026 Jul 23:2026.07.21.26358401. Preprint. [Version 1] doi: 10.64898/2026.07.21.26358401 (PMC13419489; doi:10.64898/2026.07.21.26358401)
Supplement: 1 [file NIHPP2026.07.21.26358401V1-supplement-1.pdf]

medRxiv preprint doi: https://doi.org/10.64898/2026.07.21.26358401; this version posted July 23, 2026. The copyright holder for this preprint (which was not certified by peer review) is the author/funder, who has granted medRxiv a license to display the preprint in perpetuity.
It is made available under a CC-BY 4.0 International license .

Supplementary Material: Village-level surveillance of neonatal disease with integrated real-time dashboards and quality-control in Uganda

Joseph N. Paulson[1]*, Andrew J. Whalen[1]*, Starlin Tindimwebwa[2]*, Justice Hansen[1], Davis Natukwatsa[2], Kalifuba Steven[4], Moses Ochora[3],[4], Ronnie Mulondo[1], Edith Mbabazi Kabachelor[1], Kamron Ramelmeier[5], Brian Kaaya Nsubuga[2], Philip O. Omadi[4], Joshua Magombe[13], Carmit Cohen[14], Norbert Muzahura[13], Justin Onen[13], Peter Ssenyonga[13], James R. Broach[6], Sarah U. Morton[7], Marwan Osman[1], Moses Joloba[16], Edgar Kigozi[16], Andrew Katabalwa[16], Julian Apako[16], Hilda Amutuhaire[17], John Baptist Tumuhairwe[17], Agatha Kayemba[4], Josephine Namyalo[4], Henry Masengere[4], Harriet Nambuya[8], Agatha Namutosi[8], Sophia Kasuswa[8], Emma Omo[8], Ivan Tibenkana[4], Alfred Yayi[4], Joseph Muvawala[2],[4], William Nadiope IV[15], Abraham Muwanguzi[2],[4]†, Elias Kumbakumba[9]†, Jessica E. Ericson[10]†, Steven J. Schiff[1],[11],[12]†

*Denotes equal contribution
†Denotes equal contribution

Affiliations:

[1] Department of Neurosurgery, Yale University, School of Medicine, New Haven, CT, USA
[2] Busoga Kingdom Health Department / CONRIM Uganda Field Team, Jinja, Uganda
[3] Department of Paediatrics and Child Health, Soroti University, Soroti, Uganda
[4] National Planning Authority, Kampala, Uganda
[5] University of Michigan, Ann Arbor, MI, USA
[6] Division of Newborn Medicine, Boston Children's Hospital and Department of Pediatrics, Harvard Medical School, Boston, MA, USA
[7] Institute for Personalized Medicine, Department of Biochemistry and Molecular Biology, Pennsylvania State University College of Medicine, Hershey, PA, USA
[8] Regional Referral Hospital of Jinja, Jinja, Uganda
[9] Department of Pediatrics, Mbarara University of Science and Technology, Mbarara, Uganda
[10] Division of Pediatric Infectious Disease, Pennsylvania State University College of Medicine, Hershey, PA, USA
[11] Department of Epidemiology of Microbial Diseases, Yale University School of Public Health, New Haven, CT, USA
[12] Fogarty International Center, National Institutes of Health, Bethesda, MD, USA
[13] Department of Neurosurgery, Mulago National Referral Hospital, Kampala, Uganda
[14] BGU-FOR (Food Systems, One Health & Resilience) Research Center, Ben Gurion University of the Negev, Beer Sheba, Israel
[15] Kingdom of Busoga, Bugembe, Uganda[16] Makerere University Biomedical Research Center, Kampala, Uganda[17] Department of Agricultural Production, College of Agricultural and Environmental Sciences, Makerere University, P.O. Box 7062, Kampala, Uganda

medRxiv preprint doi: https://doi.org/10.64898/2026.07.21.26358401; this version posted July 23, 2026. The copyright holder for this preprint (which was not certified by peer review) is the author/funder, who has granted medRxiv a license to display the preprint in perpetuity.
It is made available under a CC-BY 4.0 International license .

medRxiv preprint doi: https://doi.org/10.64898/2026.07.21.26358401; this version posted July 23, 2026. The copyright holder for this preprint (which was not certified by peer review) is the author/funder, who has granted medRxiv a license to display the preprint in perpetuity.
It is made available under a CC-BY 4.0 International license .

Supplementary Tables

Indicator	n (%)
Improved water source (borehole/tap, rainy)	4,988 (90.3%)
Latrine access	5,418 (98.1%)
Handwashing with soap	5,226 (94.6%)

Supplementary Table S1. Household water, sanitation and hygiene (WASH) indicators (N = 5,523). Values are n (%). Improved water source reflects borehole or tap water during the rainy season.

Substance	n	%
Water	2,228	40.3%
Nothing applied (kept dry)	2,080	37.7%
Baby powder	737	13.3%
Water and salt	424	7.7%
Alcohol / spirits	86	1.6%
Other (specified)	66	1.2%
Petroleum jelly	51	0.9%
Other	38	0.7%
Chlorhexidine (Umbi-gel)	21	0.4%
Herbs	23	0.4%

Supplementary Table S2. Substances applied to the umbilical cord stump (N = 5,523). Values are n (%); categories are not mutually exclusive where more than one substance was reported.

Water source	Rainy season n (%)	Dry season n (%)
Borehole	2,507 (45.4%)	2,784 (50.4%)
Tap water	2,481 (44.9%)	2,135 (38.7%)
Protected source	331 (6.0%)	407 (7.4%)
Shallow well	111 (2.0%)	111 (2.0%)
Unprotected well	43 (0.8%)	48 (0.9%)
Harvested rainwater	28 (0.5%)	13 (0.2%)
Other	22 (0.4%)	25 (0.5%)

Supplementary Table S3. Primary household water source by season (N = 5,523). Values are n (%) within each season.

medRxiv preprint doi: https://doi.org/10.64898/2026.07.21.26358401; this version posted July 23, 2026. The copyright holder for this preprint (which was not certified by peer review) is the author/funder, who has granted medRxiv a license to display the preprint in perpetuity.
It is made available under a CC-BY 4.0 International license .

Supplementary Figures:

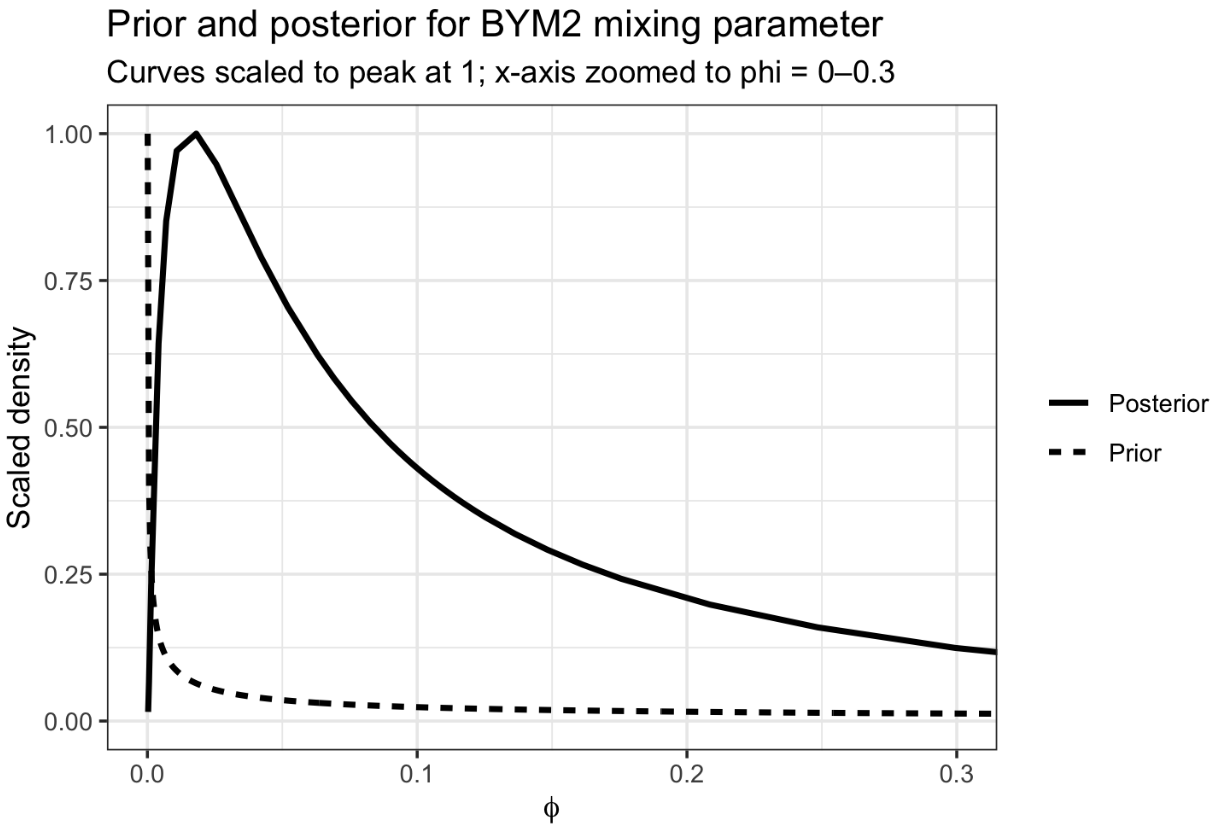

Supplementary Figure S1. Prior and posterior for the BYM-2 spatial mixing parameter. Prior (dashed) and posterior (solid) for φ, the proportion of the random-effect variance that is spatially structured. The penalized-complexity prior was weakly conservative toward the unstructured model (P(φ < 0.5) = 2/3). Curves are scaled to a common peak of 1 and the x-axis is truncated at φ = 0.3 for visibility; heights therefore reflect shape, not probability mass.

medRxiv preprint doi: https://doi.org/10.64898/2026.07.21.26358401; this version posted July 23, 2026. The copyright holder for this preprint (which was not certified by peer review) is the author/funder, who has granted medRxiv a license to display the preprint in perpetuity.
It is made available under a CC-BY 4.0 International license .

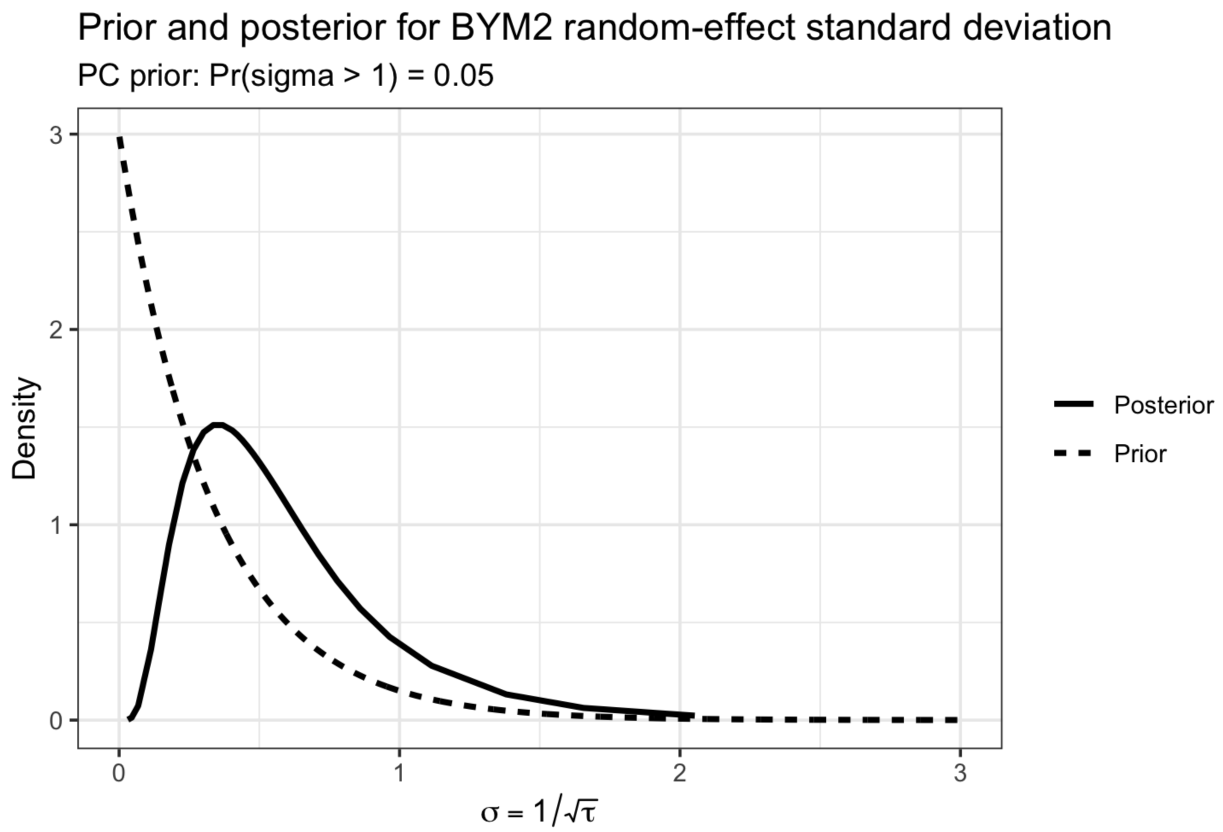

Supplementary Figure S2. Prior and posterior for the BYM-2 random-effect standard deviation. Prior (dashed) and posterior (solid) for σ = 1/√τ, the marginal standard deviation of the combined BYM-2 random effect on the log-RR scale. The penalized-complexity prior is Exponential with P(σ > 1) = 0.05. The posterior concentrated around a mode of ~0.4, well inside the prior and clearly data-informed, indicating a modest overall magnitude of village-level variation in pSBI risk.
